# Effects of Progressive Resistance Training on Post-Surgery Incontinence in Men with Prostate Cancer

**DOI:** 10.3390/jcm7090292

**Published:** 2018-09-19

**Authors:** Juhyun Park, Dong Hyun Yoon, Sangjun Yoo, Sung Yong Cho, Min Chul Cho, Ga-Young Han, Wook Song, Hyeon Jeong

**Affiliations:** 1 Department of Urology, Seoul National University College of Medicine, SMG-SNU Boramae Medical Center, Seoul 07061, Korea; urojpark@naver.com (J.P.); ebend@naver.com (S.Y.); kmoretry@daum.net (S.Y.C.); cmc1206@empal.com (M.C.C.); 2 Department of Physical Education, Institute of Sport Science, Seoul National University College of Education, Seoul 08826, Korea; ycool14@snu.ac.kr (D.H.Y.); 0104day@naver.com (G.-Y.H.); 3 Institute on Aging, Seoul National University, Seoul 08826, Korea

**Keywords:** prostatectomy, incontinence, resistance training, pelvic floor muscle

## Abstract

We evaluated the efficacy of progressive resistance training of the pelvic floor muscle for post-prostatectomy incontinence. In this prospective study, 59 patients who underwent radical prostatectomy were evaluated preoperatively. Continence was sequentially assessed within 2 weeks postoperatively, and an exercise regimen was initiated at 6- and 12-weeks. The primary outcome was continent status and the secondary outcome was changes in muscle strength and endurance after the exercise intervention. Continence was defined as no urine loss in a 1h pad test. A total of 59 patients participated in this study. Six patients dropped out of the study because of non-compliance and orthopedic problems. Of the remaining 53 patients, 31 (58.5%) achieved pad-free continence at 12 weeks postoperatively. The patients were divided into two groups based on their continence status, and no statistically significant difference was observed in age, body mass index, prostate volume, prostate-specific antigen, pathological Gleason score sum, and pathological T stage. Meanwhile, preoperative maximal urethral closure pressure and change in hip extensor muscle strength and endurance during the 12-week exercise program were significantly higher in the continent group. In multivariate analysis, change in hip extensor muscle strength was the only significant parameter predicting achievement of continence status (Odds ratio, 1.039; *p* = 0.045). The changes in hip extensor muscle strength in the current exercise program was an independent predictor of continence status after radical prostatectomy. A large-scale prospective study on the relationship between extensor muscle strength and urinary incontinence should be explored in future.

## 1. Introduction

Prostate cancer is one of the most common cancers in Western countries [1]. In South Korea, the number of prostate cancer patients has increased also rapidly in recent decades, and the disease is now one of the top five most frequent cancers occurring in men in that country [2]. These dramatic changes in prostate cancer frequency may be attributed to early diagnosis, which became possible following introduction of the prostate-specific antigen (PSA) test. Consequently, the percentage of patients with advanced and metastatic prostate cancer has decreased while that of patients with early-stage localized prostate cancer has sharply increased [3,4]. Radical prostatectomy is recognized as a standard surgical treatment for early-stage localized prostate cancer [5].

Radical prostatectomy can be classified into open laparotomy and laparoscopic surgery using conventional laparoscopic instruments or robotic instruments. Each surgical method accesses the prostate in a different way [6]. However, the primary objective of these procedures is same to remove prostate, bilateral seminal vesicles, vas, and sufficient surrounding tissues to simultaneously avoid a positive resection margin and preserve the urethral sphincter and neurovascular bundle [6,7]. To date, several surgical techniques have been developed and applied to reduce urinary incontinence especially after radical prostatectomy [8].

Urinary incontinence can arise from damage during surgery to the urethral sphincter muscle or nerves controlling it [9,10]. Stress-type incontinence, a common type of incontinence that occurs during coughing or daily activities, can increase intra-abdominal pressure, and is difficult to cure with medical treatments [10,11]. Failing to address postoperative urinary incontinence could adversely affect patients’ social and sexual lives, and act as a major factor that degrades health-related quality of life [11,12,13]. When post-prostatectomy incontinence is severe, it has been found to be associated with significantly lower patient satisfaction with the surgical treatment [13].

Kegel exercises are the most commonly practiced techniques for conservative management of post-prostatectomy incontinence [14], and various pelvic floor muscle exercise programs have been developed and spread widely for incontinence studies [15,16,17]. Those previous studies have not, however, investigated the mechanism of recovery from post-prostatectomy incontinence and have failed to make plausible assumptions [15,16,17]. There is a limit that the pelvic floor muscles is not a muscle that enters the surface and cannot be accurately measured. Thus, in current study, it was judgment that a movement to contraction an hip adductors, muscles of the pelvic region, muscles of the gluteal region, and abdominal muscles, called core muscle, could lead to stimulation and contraction in the pelvic floor muscles [18,19].

We, therefore, evaluated the efficacy of a 12-week progressive resistance training program focused on pelvic floor muscles in improving recovery of continence after radical prostatectomy, and analyzed the changes in the exercise muscle strength and endurance in relation to continence status.

## 2. Materials and Methods

This prospective study was performed for whom received radical prostatectomy for prostate cancer in a single institute from January 2015 to April 2016. The inclusion criteria were: male patient aged 60 years or older, Eastern Cooperative Oncology Group performance status of 0 or 1, and written informed consents. Exclusion criteria were as follows: adjuvant or neoadjuvant chemo-radiation therapy, severe postoperative complications, history of pelvic surgery, and diseases that could affect voiding function and limitations for the exercise program such as for patients with serious cardiovascular events or spinal or articular disease. During the periods, single surgeon (HJ) performed the radical prostatectomy. The biopsy and prostatectomy specimens were assessed by well-experienced genitourinary pathologists. Clinicopathological data, including urodynamic study, were obtained. Multichannel video urodynamic study (MMS UD-2000, Medical Measurement System, Enscheded, The Netherlands) was performed twice: once at the preoperative visit and again at 12 weeks after starting of the exercise program [20]. Changes in the maximal urethral closure pressure (MUCP), maximal cystometric capacity and detrusor pressure at maximal urinary flow (PdetQmax) were also calculated.

The Institutional Review Board (IRB) approved this study protocol. The study conformed to the tenets of the Declaration of Helsinki. Personal patient identifiers were completely removed, and data were analyzed anonymously.

### 2.1. Pelvic Floor Muscle Exercise Program

**Figure 1:**
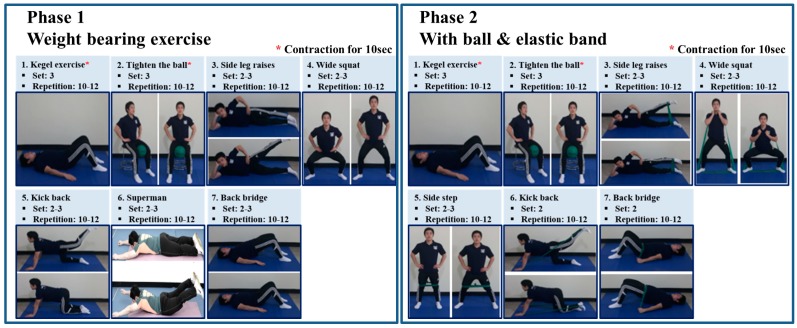
Exercise program protocol. * Contraction for 10 s.

Progressive resistance training focused on pelvic floor muscles was initiated within 2 weeks postoperatively and conducted for 12 weeks thereafter (Figure 1). This intervention recommended that Kegel-based weight-bearing exercise be started at 0–6 weeks (phase 1) postoperatively, and then elastic-band-based high-speed power training until 12 weeks (phase 2). Each session included a 10-min warm-up, 40 min of Kegel-based exercise training and 10 min of cool-down. Resting periods of 1 min were allowed between sets and 2 min between exercises. High-speed resistance training is defined as a contraction phase expected to be accomplished as quickly as possible, with a 1-s pause and an eccentric contraction exceeding 2-s [21]. High-speed resistance exercise regimens are centered on the use of elastic exercise bands, based on a previous intervention [21]. The color of the band used served to define the exercise intensity. For the power training phase, green elastic bands (very low tension) were used and participants were instructed to exercise at a rate of perceived exertion of 12–13 (‘somewhat hard’). Each high-speed resistance training exercise consisted of two to three sets of 10–12 repetitions. The exercise guideline and daily exercise checklist were created and distributed to the subjects, and the number of days of exercise was checked by bringing them in during their mid-term visits and the last visit. In addition, we performed different sets and repetitions to set individual exercise intensity. Afterwards, after an intermediate visit, the elastic band was used to increase the intensity of the exercise through tests. The exercise program followed the ACSM’s Guide to Exercise and Cancer Survivorship [22].

### 2.2. Lower Muscle Strength and Endurance

Lower limb concentric dynamic strength was measured using a HUMAC NORM isokinetic dynamometer (CSMi Solutions, Stoughton MA, USA). The hip extension/flexion and abduction/adduction peak torques of each lower limb were evaluated for the isokinetic contraction test. The participants performed the test for a maximum of three or five repetitions. Each maximal strength test was performed with an angular speed of 60°/s (velocity of 60°/s) for isokinetic muscle strength, and angular speed of 180°/s (velocity of 180°/s) for isokinetic muscle endurance measurement. The exercise was performed twice prior to testing to obtain optimal results by allowing the participants to familiarize themselves with the test [21].

### 2.3. Outcome Assessment

The primary outcome was continence status and the secondary outcome was change in muscle strength and endurance after the exercise intervention. The severity of urinary incontinence was measured using a 1 h pad test [23]. “Urinary continence” was defined as no urine loss in a 1 h pad test. Incontinence and recovery were sequentially assessed within 2 weeks postoperatively, and at 6 and 12 weeks after initiation of the exercise program.

#### Statistical Analysis

Results are presented as means ± standard deviation. Statistically significant differences of the preoperative parameters in the subgroups were analyzed using analysis of variance, chi-square, independent *t* tests and paired *t* tests. Predictors of the 12-week continence status were evaluated using logistic regression analysis. The *p*-values were two-sided, with *p* < 0.05 considered statistically significant. Statistical analysis was performed using SPSS^®^ Version 22.0 (IBM, Armonk, NY, USA).

## 3. Results

**Figure 2:**
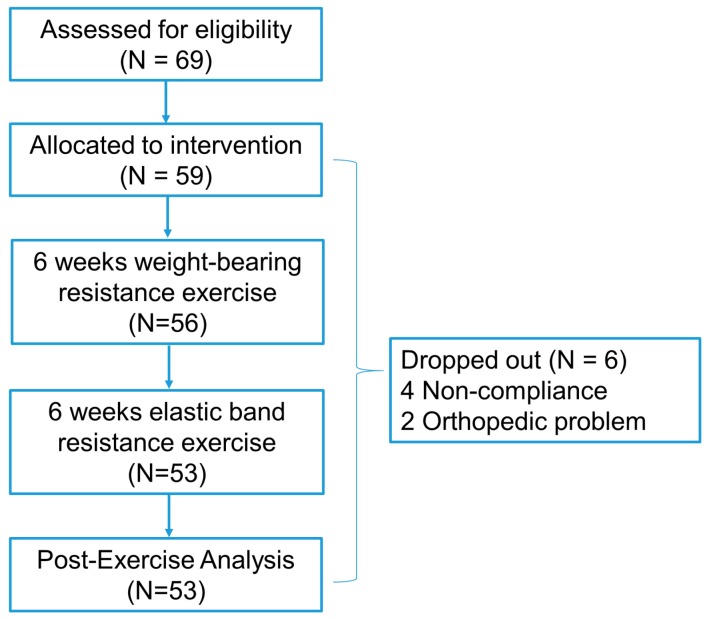
Study flow chart for 12-week exercise program.

From January 2015 to April 2016, a total of 72 patients underwent radical prostatectomy for treatment of prostate cancer at our institute. Of these, 69 were screened in accordance with the inclusion and exclusion criteria and deemed suitable candidates. The study was explained to the target group and 59 patients agreed to participate and provided their informed consent. The exercise program was initiated within 2 weeks postoperatively and was conducted for 12 weeks thereafter. Primary intermediate inspection was carried out after the first 6-week weight-bearing resistance exercise, and three patients dropped out. In the final 6-week elastic band resistance exercise, another three patients dropped out from the study. Ultimately, 53 patients completed follow-up (Figure 2). There was no adverse events related the exercise program.

In the final analysis, 58.5% (31/53) of the patients achieved pad-free status after the 12-week exercise program. When divided into continent and incontinent groups based on pad-free status (continence), there was no difference in terms of age, body mass index (BMI), prostate volume, PSA, pathological Gleason score sum, and pathological T stage between the two groups. However, preoperative MUCP was significantly higher in the continent group, though postoperative MUCP after the 12-week exercise program did not significantly differ between the groups. Furthermore, there were no meaningful changes in MUCP during the exercise program. At the 6- and 12-week evaluations, the patients in the continent group used significantly fewer pads than those in the incontinent group (Table 1).

Changes in hip extensor muscle strength and endurance during the 12-week exercise program were significantly higher in the continent group than the incontinent group, whereas the measured values of each participant’s lower limb muscle strength and endurance were comparable between the groups (Table 2).

In univariate analysis, the preoperative MUCP (odds ratio, 1.031; *p* = 0.045) and changes in the hip extensor muscle strength during the exercise program (odds ratio, 1.029; *p* = 0.010) were found to be significant variables for forecasting continence status after the 12-week exercise program. In multivariate analysis, hip extensor muscle strength was the only significant parameter predicting continence status (odds ratio, 1.039; *p* = 0.045; Table 3).

## 4. Discussion

Urinary incontinence is a major problem for men undergoing prostatectomy for treatment of their cancer, and leads to considerably reduced quality of life [12]. Therapies to reduce the incidence and severity of incontinence in these patients need to be developed and researched with regard to their efficacy. Exercise medicine interventions are particularly attractive given their relatively rapid and inexpensive implementation, and lack of side effects such as those inherent in any pharmaceutical approach [10,24]. Therefore, a recently published study protocol was planned using different times of exercise initiation, exercise durations, and combinations of exercise programs [15,16,17]. Centemero and his colleagues published the early starting of pelvic floor muscle exercise before radical prostatectomy could improve early continence. In that study, physiotherapist helped the pelvic floor muscle exercise twice per week [15]. Parekh et al revealed the formal education and instruction of pelvic floor muscle exercise showed significant benefit to achieve continent status [16]. Tienforti et al showed that preoperative biofeedback combined with a postoperative pelvic floor muscle exercise would be more effective treatment strategy in improving recovering of incontinence [17].

Thus far, previous studies have not investigated in detail the mechanism how pelvic floor muscle exercises can help patients recover from urinary incontinence [15,16,17]. Because the pelvic floor muscles could not be accurately measured and specifically trained, lower limb exercise of current exercise program to contract an hip adductors, muscles of the pelvic region, muscles of the gluteal region, and abdominal muscles might be the clues for the recovering mechanism from post-prostatectomy incontinence [18,19]. The changes of hip joint muscle strength or endurance were not simply the change of pelvic floor muscle strength or endurance. However, the continent group, the change of hip extensor muscle strength and endurance were definitely significant between the continent and incontinent groups. Ultimately, it could be reasonable to hypothesize that the change in hip extensor muscle strength is related to achievement of continence status and to design another exercise program to focus the reinforcement of hip extensor muscle.

Interestingly, in the present study, the rate of achieving continence did not differ by degree of participation in exercise therapy. When patients were classified into an active and a passive group according to their degree of participation in the training program based on a threshold of 50% participation, 27 patients were classified into the former and 26 in the latter. Contrary to expectations, the rates of continence in the active and passive groups were 55.6% and 61.5%, respectively, and the difference was not statistically significant. These results suggested that performing the exercise correctly may be more important in strengthening the essential muscles associated with recovery from urinary incontinence.

This study was the first to reveal that change in hip extensor muscle strength and endurance could be related to achievement of continence, and this novel finding can be applied clinically in various ways. First, a newly developed exercise method can be provided to reinforce hip extensor muscle in patients with incontinence after radical prostatectomy. Furthermore, a new exercise method can be adopted to treat stress urinary incontinence in women and incontinence in men after transurethral prostatectomy such as holmium laser enucleation of the prostate [25].

In recent reviews, pelvic floor muscle exercise for urinary incontinence after radical prostatectomy was considered an effective treatment option for accelerating restoration of continence [9,10,11]. Similarly, preoperative pelvic floor muscle exercises were reported to potentially aid faster return to continent status at 3 months postoperatively, though there were no significant differences in continence rates at 6 months postoperatively [24]. Although there was no difference in the long-term continence rates, the results remain meaningful if the exercise program enables faster recovery from urinary incontinence. Early recovery from urinary incontinence is also central issue for patients who undergo prostatectomy [11,24,26].

The severity of urinary incontinence was measured using a 1h pad test, which was standardized as the specific protocol for evaluating urinary incontinence [23]. Other pelvic floor muscle exercise studies assessed the degree of incontinence based on a 24 h pad test or the number of pads used in a day [15,16,17]. In present study, self-reported numbers of pads used per day was not matched with continent status. Some patients with a negative 1h pad test used pads in real life, while other patients with a positive 1 h pad test used no pads. The patient’s individual character influence the number of pads used daily.

Some studies have tried to improve recovery from incontinence by using a meticulous minimally invasive technique to preserve the neurovascular bundles during surgery [26]. Recovery of urinary incontinence after radical prostatectomy is also expected to be faster if applying posterior musculofascial reconstruction during the surgery [27]. In the present study, a single surgeon (HJ) performed all the radical prostatectomy procedures. Two operative methods were used: robot-assisted laparoscopic radical prostatectomy and retropubic radical prostatectomy. Posterior reconstruction was performed during each surgical technique. The rate of continence achievement was not related to the surgical method used.

The present study showed that appropriate exercise intervention after radical prostatectomy in patients with prostate cancer has great merit for recovery of general physical and mental condition [11,12,13]. The study does have some limitations. For one, it was a prospective study, but it was not a randomized controlled study. Additionally, the sample size of patients was relatively small, and 10% of the initial study population stopped participating. Finally, a single operator performed the radical prostatectomy, but two different surgical methods were applied.

Nevertheless, the results of the present study are meaningful, as we reported the effect of pelvic exercise therapy on change in hip joint muscle strength and found the exercise program yielded significant results. Future studies on urinary incontinence should develop more structured and efficient exercise methods that can reinforce hip extensor muscle.

## 5. Conclusions

The total continence rate in patients who underwent radical prostatectomy was 58.5% following a supervisor-assisted 12-week progressive resistance training program. Change in hip extensor muscle strength during the exercise program was an independent predictor of post-prostatectomy continence status. The relationship between hip extensor muscle and urinary incontinence should be explored in future studies.

## Figures and Tables

**Table 1:** Patient characteristics according to the achievement of post-exercise continent status.

	Continent Group (*n* = 31)	Incontinent Group (*n* = 22)	*p*-Value
Age (years)	68.2 ± 6.1	68.3 ± 5.1	0.899
Preoperative BMI (kg/m^2^)	24.8 ± 2.8	26.6 ± 9.0	0.289
Post-exercise 12-weeks BMI (kg/m^2^)	24.5 ± 2.8	24.7 ± 3.3	0.827
Prostate volume (mL)	36.4 ± 19.1	37.8 ± 23.5	0.827
PSA (ng/dL)	23.6 ± 42.1	24.3 ± 42.8	0.953
Op methods			0.601
RRP	17	12	
RALP	14	10	
Pathology Gleason Score Sum			0.812
6	5	4	
7 (3 + 4)		7	5
7 (4 + 3)	8	8	
8	7	4	
9	4	1	
Pathologic T stage			0.219
T2	20 (64.5%)	11 (50.0%)	
T3	11 (35.5%)	11 (50.0%)	
Participation rate (%)	42.0 ± 38.7	50.4 ± 36.0	0.428
Preoperative MUCP (cmH_2_O)	78.2 ± 18.8	64.8 ± 25.3	0.036
Preoperative MCC (mL)	355.3 ± 133.1	339.4 ± 139.2	0.685
Preoperative PdetQmax (cm H_2_O)	55.1 ± 21.0	48.7 ± 20.2	0.285
Post-exercise 12-week MUCP (cm H_2_O)	56.1 ± 25.3	53.6 ± 27.0	0.739
Post-exercise 12-week MCC (mL)	339.5 ± 89.7	297.1 ± 77.3	0.085
Post-exercise 12-week DetQmax (cm H_2_O)	38.5 ± 18.5	30.6 ± 15.6	0.123
Change of MUCP (cm H_2_O)	24.4 ± 29.4	12.9 ± 30.6	0.199
Post-exercise 6-week pad (g)	14.2 ± 45.6	18.7 ± 23.8	0.676
Post-exercise 6-week pad number	1.67 ± 2.1	4.2 ± 5.8	0.034
Post-exercise 12-week pad (g)	0	14.7 ± 25.1	0.002
Post-exercise 12-week pad number	0.4 ± 0.5	2.14 ± 2.4	<0.001

BMI, Body mass index; PSA, prostate-specific antigen; RRP, retropubic radial prostatectomy; RALP, robot-assisted laparoscopic radical prostatectomy; MUCP, Maximal urethra closure pressure; MCC, maximal cystometric capacity; PdetQmax, detrusor pressure at maximal urinary flow in pressure-flow study.

**Table 2:** Change of pelvic muscle strength and endurance according to the achievement of post-exercise continent status.

	Continent Group (*n* = 31)		Incontinent Group (*n* = 22)		*p*-Value
	Pre	Post	Pre	Post	
**Isokinetic 60°/s peak torque/Bodyweight, muscle strength**					
**Hip Extensor (Nm)**					
Pre/Post exercise muscle strength	119.5 + 41.7	145.9 + 41.3	138.0 + 67.5	139.6 + 55.4	
Change of muscle strength	22.3 ± 39.7		−9.5 ± 32.4		0.005
**Hip Flexor (Nm)**					
Pre/Post exercise muscle strength	78.1 + 25.9	92.2 + 26.1	85.0 + 34.1	90.6 + 19.3	
Change of muscle strength	11.5 ± 26.9		1.4 ± 27.2		0.204
**Hip Abductor (Nm)**					
Pre/Post exercise muscle strength	75.5 + 19.4	87.5 + 28.2	70.5 + 24.7	71.9 + 32.7	
Change of muscle strength	12.0 ± 26.4		10.2 ± 26.3		0.811
**Hip Adductor (Nm)**					
Pre/Post exercise muscle strength	109.1 + 37.7	122.2 + 40.5	116.1 + 43.5	130.7 + 41.5	
Change of muscle strength	21.7 ± 48.4		3.9 ± 31.0		0.153
**Isokinetic 180°/s peak torque / Bodyweight, muscle endurance**					
**Hip Extensor (Nm)**					
Pre/Post exercise muscle endurance	85.3 + 65.4	106.9 + 38.8	102.5 + 53.9	105.1 + 42.6	
Change of muscle endurance	18.8 ± 43.6		−5.6 ± 33.1		0.038
**Hip Flexor (Nm)**					
Pre/Post exercise muscle endurance	65.1 + 65.4	62.1 + 17.4	58.8 + 25.2	66.5 + 18.1	
Change of muscle endurance	−5.2 ± 61.9		4.2 ± 20.1		0.517
**Hip Abductor (Nm)**					
Pre/Post exercise muscle endurance	63.0 + 21.0	70.9 + 24.0	64.8 + 23.0	66.5 + 18.1	
Change of muscle endurance	7.9 ± 25.6		3.1 ± 14.3		0.449
**Hip Adductor (Nm)**					
Pre/Post exercise muscle endurance	79.3 + 36.3	89.3 + 29.9	82.4 + 30.4	89.1 + 36.2	
Change of muscle endurance	10.0 ± 32.2		5.7 ± 27.7		0.624

**Table 3:** Logistic regression analysis to predict post-exercise 12 week continent status.

	Univariate		Multivariate	
	*p*-Value	OR (95% CI)	*p* Value	OR (95% CI)
Age	0.897	0.994 (0.901–1.096)	0.686	0.965 (0.811–1.148)
BMI	0.354	0.943 (0.833–1.067)	0.800	0.963 (0.719–1.289)
Prostate volume	0.822	0.997 (0.968–1.026)	0.454	0.984 (0.942–1.027)
Pathologic T stage	0.293	0.550 (0.181–1.675)	0.773	1.369 (0.162–11.535)
Operative Methods (RRP vs. RALP)	0.983	0.988 (0.330–2.962)	0.553	1.811 (0.280–11.703)
MUCP	0.045	1.031 (1.001–1.061)	0.097	1.046 (0.992–1.103)
Change of hip extensor muscle strength (Nm)	0.010	1.029 (1.007–1.053)	0.045	1.039 (1.001–1.080)

BMI, Body mass index; RRP, retropubic radial prostatectomy; RALP, robot-assisted laparoscopic radical prostatectomy; MUCP, Maximal urethra closure pressure.
